# Circular RNA MAPK4 (circ-MAPK4) inhibits cell apoptosis via MAPK signaling pathway by sponging miR-125a-3p in gliomas

**DOI:** 10.1186/s12943-019-1120-1

**Published:** 2020-01-28

**Authors:** Jiehua He, Zuoyu Huang, Mingliang He, Jianyou Liao, Qianqian Zhang, Shengwen Wang, Lin Xie, Leping Ouyang, H. Phillip Koeffler, Dong Yin, Anmin Liu

**Affiliations:** 1 Guangdong Provincial Key Laboratory of Malignant Tumor Epigenetics and Gene Regulation, Sun Yat-Sen Memorial Hospital, Sun Yat-Sen University, Guangzhou, 510120 China; 2 Research Center of Medicine, Sun Yat-Sen Memorial Hospital, Sun Yat-Sen University, 107 Yan-Jiang Xi Road, Guangzhou, 510120 China; 3 Department of Neurosurgery, Sun Yat-Sen Memorial Hospital, Sun Yat-Sen University, 107 Yan-Jiang Xi Road, Guangzhou, 510120 China; 4 Vascular Biology Research Institute, School of Life Sciences and Biopharmaceutics, Guangdong Pharmaceutical University, Guangzhou, 510006 China; 5 Division of Hematology/Oncology, Cedars-Sinai Medical Center, School of Medicine at University of California Los Angeles, Los Angeles, California USA

**Keywords:** Circ-MAPK4, P38/MAPK signaling pathway, MiR-125a-3p, Gliomas, Apoptosis

## Abstract

**Background:**

Recent evidences have shown that circular RNAs (circRNAs) are frequently dysregulated and play paramount roles in various cancers. circRNAs are abundant in central nervous system (CNS); however, few studies describe the clinical significance and role of circRNAs in gliomas, which is the most common and aggressive primary malignant tumor in the CNS.

**Methods:**

A bioinformatics analysis was performed to profile and screen the dyregulated circRNAs during early neural development. Quantitative real-time PCR was used to detect the expression of circ-MAPK4 and target miRNAs. Glioma cells were transfected with circ-MAPK4 siRNAs, then cell proliferation, apoptosis, transwell assays, as well as tumorigenesis and TUNEL assays, were performed to examine effect of circ-MAPK4 in vitro *and vivo*. Biotinylated-circ-MAPK4 probe based pull-down assay was conducted to confirm the relationship between circ-MAPK4 and miR-125-3p.

**Results:**

In this study, we identified a circRNA, circ-MAPK4 (has_circ_0047688), which was downregulated during early neural differentiation. In gliomas, circ-MAPK4 acted as an oncogene, was inversely upregulated and linked to clinical pathological stage of gliomas (*P* < 0.05). Next, we verified that circ-MAPK4 promoted the survival and inhibited the apoptosis of glioma cells in vitro *and* in vivo. Furthermore, we proved that circ-MAPK4 was involved in regulating p38/MAPK pathway, which affected glioma proliferation and apoptosis. Finally, miR-125a-3p, a miRNA exhibited tumor-suppressive function through impairing p38/MAPK pathway, which was increased by inhibiting circ-MAPK4 and could be pulled down by circ-MAPK4. Inhibition of miR-125a-3p could partly rescue the increased phosphorylation levels of p38/MAPK and the elevated amount of apoptosis inducing by knockdown of circ-MAPK4.

**Conclusions:**

Our findings suggest that circ-MAPK4 is a critical player in glioma cell survival and apoptosis via p38/MAPK signaling pathway through modulation of miR-125a-3p, which can serve as a new therapeutic target for treatment of gliomas.

## Background

Glioma is the most common and fatal primary nervous system tumor [1, 2], characterized by heterogeneous genetic molecular aberrations [3]. It can be sorted into four grades according to the WHO criteria 2007 [4], among which glioblastoma multiforme (GBM) belonging to WHO grade IV which is the most aggressive form with repeated recurrence. The median survival rate of GBM patients is only 14.6 months, even under standard advanced surgery, temozolomide-based chemotherapy and radiotherapy [5, 6]. Thus, exploring the possible molecular mechanisms involved in gliomas progression is urgently needed to improve treatment of gliomas, especially GBM.

Circular RNAs (circRNAs) are a type of newly classified noncoding RNAs (ncRNAs), which contain covalently closed loop structures without 5′ to 3′ polarity and polyadenylated tail [7, 8]. A series of studies have reported that circRNAs are conserved in mammals and specifically expressed in some cell types and developmental stages [9–11]. Studies have claimed that circRNAs may play an important role in different diseases and pathophysiological processes [12–14]. Bioinformatics analysis showed that dysregulated circRNAs might be associated with tumorigenesis and development of GBM [15]. Recently, eIF4A3-induced circMMP9 was an important oncogene in cell proliferation and metastasis of GBM through regulation of the miR-124 signaling pathway, which could provide pivotal potential therapeutic targets for the treatment of GBM [16]. Hence, elucidation of the molecular mechanisms underlying circRNAs may lead to promising therapeutic candidates; however, the mechanisms and functions of circRNAs are not completely clear in gliomas.

In glioma, proteins such as Sox2, Bmi1, hairy and enhancer split (Hes) play crucial roles in neural development and promote self-renewal and proliferation in glioma [17]. In this study, we show that circ-MAPK4 (has_circ_0047688), which is downregulated after neural differentiation in an induction model by stimulation with retinoic acid [18], but is upregulated in gliomas tissues and cell lines. We hypothesized that circ-MAPK4 may affect gliomas progression. Our experimental results suggested that circ-MAPK4 resumes survival and inhibits apoptosis in glioma via p38/MAPK signaling pathway. Furthermore, we found that circ-MAPK4 acts as a specific sponge for miR-125a-3p, which is reported to be a tumor-suppressor in glioma [19]. The role of circ-MAPK4 in inhibiting the apoptosis of glioma cells was also shown in vivo.

## Methods

### Clinical samples

All glioma tissues (*n* = 30, random World Health Organization [WHO] grade) were collected from patients who were underwent surgical resection at the Department of Neurosurgery, Sun Yat-Sen Memorial Hospital, Sun Yat-Sen University from January 2016 to October 2017, including 11 females and 19 males (age range, 24–70 years; median, 47 years). None of the patients was treated with either chemotherapy or radiation prior to surgery. Moreover, 8 normal brain tissues from patients who were non-glioma diseases were resected at the Department of Neurosurgery, Sun Yat-Sen Memorial Hospital, SunYat-Sen University from January 2016 to October 2017, including 5 females and 3 males. After resection, all samples were snap-frozen in liquid nitrogen immediately and stored at − 80 °C. These tissues were approved by medical ethics committee. Detailed information is presented in Table 1.

**Table 1:** Clinicopathological features of 30 glioma patients and the expression of circ-MAPK4

Parameters	Group	Cases	Circ-MAPK4 expression				*P*-value
			Low	%	High	%	
Gender	Male	19	10	52.6	9	47.3	0.919
	Female	11	6	54.5	5	0.45	
Age at surgery	< 30	9	6	66.7	3	33.3	0.855
	> 30	21	10	47.6	11	52.3	
Pathological stage	WTO:I~II	7	7	100	0	0	**0.044**
	WTO:III~IV	23	9	39.1	14	60.8	
Tumor size (maximum diameter in MRI)	> = 30 mm	14	8	57.1	6	42.8	0.513
	< 30 mm	16	8	50	8	50	
Tumor necrosis	Yes	11	4	36.3	7	0.63	0.156
	No	19	12	63.1	7	36.8	
Total		30	15	50	15	50	

### Cell culture

U138, U373 and U87 glioma cell lines were purchased from American Type Culture Collection (ATCC, USA). Cells were cultured in high-glucose Dulbecco’s modified Eagle’s Medium (DMEM; Invitrogen-Gibico), containing 10% fetal bovine serum (FBS; Invitrogen-Gibico), 1% penicillin/streptomycin (Invitrogen) at 37 °C in a humidified chamber with 5% CO_2_. Glia cell line (HA1800) was purchased from ATCC and was cultured in Astrocyte Medium (ScienCell, #1801), which containing, 10% FBS, 10% astrocyte growth supplement and 1% penicillin/streptomycin at 37 °C in a humidified chamber with 5% CO_2_.

### RNA extraction, treatment with RNase R

Total RNAs were extracted from cell lines and tissues using Trizol (Invitrogen) according to the manufacturer’s instructions. RNase R treatment was carried out for 30 min at 37 °C using 3 U/mg of RNase R (Epicenter Technologies, RNR07250). For RT–PCR, the treated 400 ng RNA was directly reverse transcribed using PrimeScript™ RT Master Mix (Takara, RR036A) containing random and oligo (dT) primer. PCR was then conducted using GoTaq® Green Master Mix (Promega, M712) according to manufacturer’s instructions along with PCR control. Products were separated on a 2% agarose gel and visualized with GelRed. Specific primers used in PCR are listed in Additional file 1: Table S1.

### Quantitative real-time PCR (qPCR)

To quantify circRNA, mRNA, and miRNA, qPCR analyses were performed using LightCycler® 480 SYBR Green I Master (Roche, 04707516001) using the LightCycler 480 fluorescence quantitative PCR instrument (Roche). PCR conditions were as follows: pre-incubation at 95 °C for 5 min, followed by 40 cycles of amplification at 95 °C for 10 s, 60 °C for 20 s and a final elongation at 72 °C for 15 s. GAPDH or U6 was used as the internal control, and the relative expression of target genes was calculated by 2^-ΔΔCt^ method. Primers are listed in Additional file 1: Table S1.

### Nucleocytoplasmic separation

Total of 1 × 10^7^ cells were resuspended in 1 ml RLN buffer (50 mM Tris-HCl pH 7.4, 0.14 M NaCl, 1.5 mM MgCl_2_, 0.5% IGEPAL CA-630, 1 mM DTT) for 5 min incubation on ice, followed by homogenization. Cytoplasmic fraction was the supernatant after centrifugation. Pellet were resuspended in 1 ml RSB buffer (0.25 M sucrose,10 mM Tris-HCl pH 7.4,10 mM NaCl,3 mM MgCl_2_, 1 mM DTT, 0.5 mM PMSF), followed by homogenization and centrifugation to clean out the residual cytoplasmic faction. Finally, the preliminary nuclear fraction was resuspended in 3 ml RSB buffer, followed by adding 3 ml 2 M RSB (2 M sucrose,10 mM Tris-HCl pH 7.4, 10 mM NaCl,3 mM MgCl_2_, 1 mM DTT, 0.5 mM PMSF) and was centrifugated at 30,000 g for 45 min. Total RNA was extracted from nuclear and cytoplasmic fractions using RNA Trizol reagent.

### Oligonucleotide transfection

siRNAs were synthesized by Gene Pharma (Shanghai, China). Sequences used are listed in Additional file 1: Table S2. Transfection was carried out using RNAiMAX (Life Technologies, 13,778–150) according to the manufacturer’s instructions.

### Cell proliferation assays

According to the procedure described above, U138 and U373 cells transfected with si-circ-MAPK4 or circ-MAPK4 overexpression vector were trypsinized and reseeded in 96-well plates (1 × 10^3^ cells per well), and cell viability was assessed by CCK-8 assays (APExBio, USA). The absorbance of each well was read at a wavelength of 450 nm on a SPARK 10 M spectrophotometer (Tecan, Austria). For cell colony formation assays, the treated U138 and U373 cells were trypsinized and placed in 6-well plates (1 × 10^3^ cells per well). The cells were dispersed evenly by slightly shaking the dishes and were incubated at 37 °C with 5% of CO2 for 7 days until the visible colonies appeared. Media were moved and the cells were carefully washed twice with PBS. After being fixed with methanol for 15 min, cells were stained with dyeing solution containing 0.1% crystal violet for 15 min. Clones with more than 50 cells were counted with an ordinary optical microscope and the colony formation rate was calculated with the following formula: Plate clone formation efficiency = (number of clones/number of cells inoculated) × 100%.

### Apoptosis assays

Apoptosis assays were performed using Annexin V-FITC Apoptosis Detection Kit (Invitrogen, Carlsbad, Calif, USA). In brief, either transfected U138 or U373 cells (4 × 10^5^ per well) were starved in FBS-free medium for 12 h, trypsinized and treated with binding buffer with 5 μl Annexin V-FITC and 10 μl PI (incubated at room temperature for 20 min in the dark). Cell apoptosis was analyzed by flow cytometry (BD Biosciences, San Jose, CA, USA).

### Cell invasion assays

For transwell invasion assays, a 24-well transwell chamber (Costar, USA) with precoated Matrigel was used to detect cell invasive ability according to the manufacturer’s protocol. Cells suspended in 0.2 ml serum-free medium (1 × 10^5^/well) were added to the upper chambers, and media supplemented with 10% FBS was applied to the lower chambers. After incubating the cells for 15 h (for U138) and 24 h (for U373) at 37 °C; and 5% CO_2_, cells that invaded to the lower membrane surface were fixed with 4% paraformaldehyde and stained with 1% crystal violet in PBS. Invaded cells were counted in five randomly selected fields. Three independent experiments were performed in triplicate.

### Western blot assay

U138 and U373 cells were harvested and extracted in RIPA lysis buffer, pelleted by centrifugation at 12,000 g at 4 °C for 20 min. Cell extracts were boiled in loading buffer and equal amount of cell extracts were separated on 10% SDS-PAGE gels. Proteins were transferred to PVDF membrane for two hours at 300 mA. After blockaded with 5% milk, the membrane were incubated with the primary antibodies: anti-cleaved-caspase 9 (a2324, Abcam, Cambridge, MA, USA), anti-cleaved-caspase 7 (ab2323, Abcam), anti-cleaved-caspase 3 (ab2303, Abcam), anti-PARP1 and anti-cleaved-PARP1 (ab137653, Abcam), anti-p38 antibody [E229] (ab170099, Abcam), anti-p38 (phospho T180 + Y182) antibody (ab4822, Abcam), anti-MAPK6/ERK-3 [EP1720Y] (ab53277, Abcam), anti-ERK1 (phospho T202) + ERK2 (phospho T185) [EPR19401] (ab201015, Abcam), anti-JNK (66210–1-Ig, Proteintech), anti-CREB1 (12208–1-AP, Proteintech), anti-β-actin (ab8227, Abcam) and anti-GAPDH (ab153802, Abcam), each was diluted at a ratio of 1:1000 and incubated overnight at 4 °C, followed washing with PBS/T (Phosphate Buffer Solution with 0.05% Tween20). Then membranes were incubated with horseradish peroxidase-linked secondary antibody at room temperature for 1 h. Immunoreactive bands were displayed using ECL kit (Millipore), GAPDH or β-actin was used as loading control.

### Pull-down assay

Biotinylated probes, which are reversed complementary to circ-MAPK4 black-splice junction sequences (circ-MAPK4 probe) or random sequences (oligo probe) were designed and synthesized by Sangon Biotech (Shanghai, China) (Additional file 1: Table S3). Pull-down assays were performed according to the protocol. Briefly, glioma cells were harvested, crosslinked, lysed, and sonicated. Circ-MAPK4 probe and control oligo probe were incubated with cell lysates at 37 °C overnight. After hybridization, C1 magnetic beads (Life Technologies) which have been precleaned, were added to the lysates and incubated at 37 °C for 1 h to generate circRNA-probe-beads complex. After using washing buffer, the RNA (bound to the beads) was eluted, extracted by Trizol (Takara) followed by real-time PCR.

### Animal studies

Four-week-old male BALB/c nude (nu/nu) mice were purchased from the Nanjing Biomedical Research Institute of Nanjing University. These were housed under barrier conditions and maintained on a 12-h light/12-h dark cycle, with food and water supplied ad libitum. Animal studies were approved by the Institutional Animal Care and Use Committee of Sun Yat-Sen Memorial Hospital.

For the ectopia xenograft nude mouse model, totally 10 mice (*n* = 5 each group) were subcutaneous implanted with the stable circ-MAPK4 knockdown U138 cells (5 × 10^6^) or parent control U138 cells suspended in 100 μl PBS. Tumor volume was measured every 7 days, and animals were sacrificed 35 days after injection and tumors were collected to measure the final tumor volume, which was calculated using: volume (mm3) = length × width^2^/2.

For the orthotopic xenograft nude mouse model, the bregma was identified and a small bur hole was created 2.0 mm lateral to it. Either stable circ-MAPK4 silenced or parent control U87 cells suspension (1 × 10^6^, in 10 μl PBS) were implanted via a Hamilton syringe exactly 3.5-mm deep to the dura of the brains. Both the stable U87 cells contained a GFP marker. In vivo tumorigenicity was monitored by In-Vivo FX Pro system (Bruke Corporation) every 5 days from 30 days post-implantation, and mice were sacrificed when showing clinical symptoms. Mice brains were subjected to HE staining as indicated and were scanned by TussueFAXS Cytometry (TissueGnostics Corporation). The largest tumor areas in the serial sections were identified. Survival analysis was calculated by Kaplan-Merier curve.

### TUNEL assay

Terminal deoxynucleotidyl transferase (TdT) dUTP Nick-End Labeling (TUNEL) assay has been designed to detect apoptotic glioma cells that undergo extensive DNA degradation during the late stages of apoptosis. Tumors in nude mice (the model mentioned above) were fleshly removed, frozen in Tissue-Tek OCT Compound (Miles, Elkhart, IN) by immersion in a 2-methylbutane bath on dry ice. 5 μm section were stained by terminal deoxynucleotidyl transferase-mediated dUTP-biotin nick end labeling (TUNEL) method according to the instruction, followed by using an apoptosis in situ detection kit (Wako Pure Chemical, Osaka, Japan). TUNEL-positive cells labeled with FITC were imaged by fluorescence microscopy using excitation at 488 nm and emission at 530 nm. After randomly selecting three fields per section, the observer counted FITC-positive cells. Ratios of TUNEL-positive cells compared to total cells are shown. The results were summarized and presented in bar graph as means ± standard deviation (SD).

### Statistical analysis

Data were analyzed using Student’s t-test and ANOVA using SPSS 17.0 software (SPSS, Chicago, IL, USA). Each experiment was repeated at least three times. All results are summarized and presented as means ± standard deviation (SD). A *P* value less than 0.05 was considered statistically significant. To analysis data downloaded from Rajewsky N.’s research, we used the cluster 3.0 with complete linkage and centered Pearson correlation to perform hierarchical clustering. Before performing unsupervised hierarchical clustering, normalized and log_2_-scaled signal ratios were centered on the median.

## Results

### Circ-MAPK4 is highly expressed in early neural stage and glioma tissues, and data were correlated with clinic pathological parameters

**Fig. 1:**
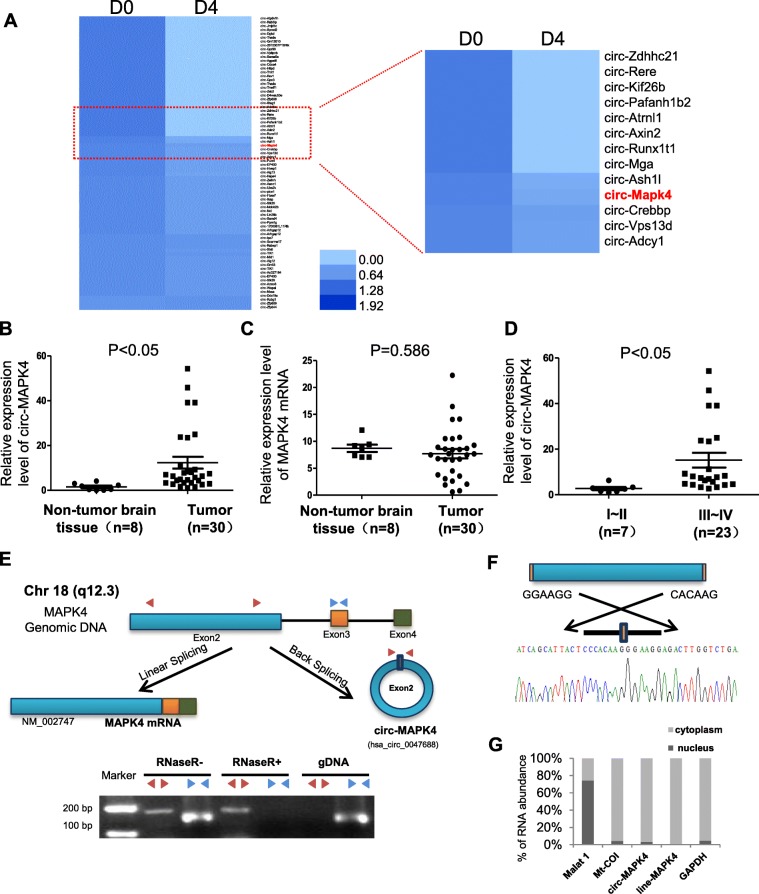
Characterization of circ-MAPK4 in human neural embryoid body and gliomas. (**a**) Heat map of microarray data which identified aberrantly expressed circRNAs in the first day (D0) and the 4th day (D4) upon inducing neural differentiation of murine P19 embryonic carcinoma (EC). (**b**) circ-MAPK4 is upregulated in glioma tissues compared with normal brain tissues (*P* < 0.05). (**c**) The level of MAPK4 mRNA did not show significant difference between glioma tissues and normal brain tissues (*P* > 0.05). (**d**) circ-MAPK4 is highly expressed in gliomas patients with advanced stages III + IV compared with I + II (*P* < 0.05). (**e**) Upper panel: Schematic representation of circ-MAPK4 (hsa_circ_0047688) formation. Divergent (red) and convergent (blue) primers were designed to amplify the back-splicing and linear products. Lower panel: Compared to the MAPK4 mRNA, RT-PCR revealed that circ-MAPK4 resistant to the RNase R, which also remain 80–90% after digested by RNase R. No product was amplified to genomic DNA using divergent primers. (**f**) The back-splice junction site of circ-MAPK4 was validated by RT–PCR followed by Sanger sequencing. (**g**) By the Nucleocytoplasmic separation experiment, circ-MAPK4 was identified in the cytoplasm mainly, which is consistent with the MAPK4 mRNA. MALAT and Mt-Coi is the marker of nucleus and cytoplasm, respectively. GAPDH was used as control

According to Rajewsky N.’s research of inducing mouse P19 embryonic carcinoma (EC) neural differentiation by stimulation with retinoic acid [18], a large amount of circRNAs were differentially expressed on the 4th day of induction which could be regarded as early neural differentiation. Our bioinformatics analysis focused on the downregulated circRNAs during early stage of neural differentiation and revealed that circ-MAPK4 (hsa_circ_0047688) was significantly decreased on the 4th day after stimulation (D4) compared with non-stimulation (D0) (Fig. 1a). Considering the dedifferentiation status of glioma, circ-MAPK4 was found (Fig. 1b), but not the MAPK4 mRNA (Fig. 1c), to be significantly overexpressed in glioma tissues compared with normal brain tissues as measured by qPCR using divergent primers. Moreover, upregulation of circ-MAPK4 occurred in GBM by MiOncoCirc database (Additional file 2: Figure S1A). For the others circRNAs found in the neural differentiation model, 9 circRNAs expression profile were examined in our glioma tissues, but no more significantly overexpression were found in glioma tissues like circ-MAPK4 (Additional file 2: Figure S1B). Moreover, in our validation cohort, circ-MAPK4 was elevated in patients with advanced stages of gliomas (III + IV vs I + II, *P* < 0.05) (Table 1, Fig. 1d). These results above suggested an oncogenic role for circ-MAPK4 in gliomas tumorigenesis.

### Circ-MAPK4 is resistant to RNase R digestion and predominantly located in the cytoplasm

circ-MAPK4 arises from the MAPK4 gene and consists of the head-to-tail splicing of exon 2 (1416 bp) as reported in circBase. We found that circ-MAPK4 is highly conservative among mammals through performing multiple alignments with sequences of circ-MAPK4 (Additional file 3: Figure S2A) and 10 flanking sequences of circ-MAPK4 (Additional file 3: Figure S2B).To verify the exon 2 of MAPK4 gene formed an endogenous circRNA, we designed divergent and convergent primers that specifically amplified the back-spliced and canonical forms of MAPK4 (Fig. 1e, upper panel). circ-MAPK4 was detected by RT-PCR with divergent primers (red), which was resistant to the digestion by RNase R. In contrast, convergent primers (blue) amplified the MAPK4 mRNA (exon3), which disappeared after RNase R digestion. Furthermore, no genomic DNA was amplified using the divergent primers, eliminating artifacts caused by genomic rearrangement (Fig. 1e, lower panel). Next, Sanger sequencing was used to confirm the black-spliced junction point of circ-MAPK4 (Fig. 1f). Expression ratio of circ-MAPK4 in cytoplasmic and nuclear fractions was measured by qPCR after nucleocytoplasmic separation. Approximately 96% of circ-MAPK4 transcripts were found in the cytoplasm (Fig. 1g).

### Circ-MAPK4 behaves as an oncogene in glioma cells

**Fig. 2:**
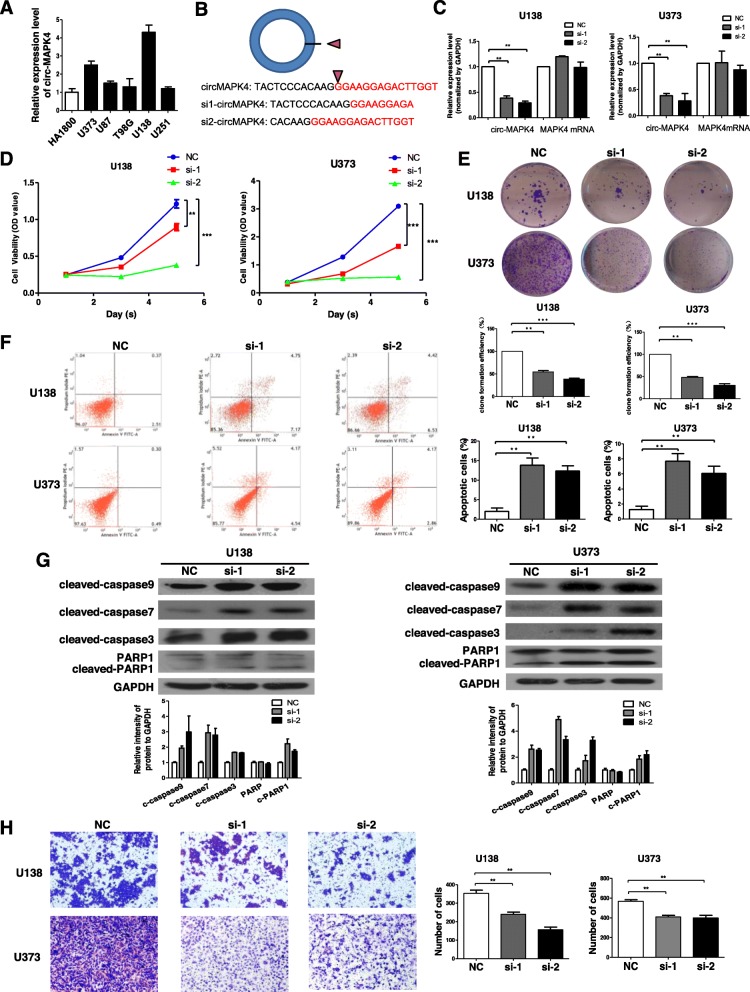
circ-MAPK4 behaved as an oncogene in glioma cells. (**a**) Circ-MAPK4 is also highly expressed in glioma cell lines, especially in U138 and U373, when compared to gial cell line (HA1800). (**b**) Schematic representation of target sequences of siRNAs which specially silence circ-MAPK4. (**c)** The silence efficiency of siRNA targeting to circMAPK4 was analyzed by qPCR after transfected for 48 h in either U138 or U373 cells. (**d**) CCK-8 assays were performed to test survival of U138 and U373 cells after silencing of circ-MAPK4. (**e**) Colony formation assays were performed to test survival of U138 and U373 cells after silencing of circ-MAPK4. (**f**) Apoptosis assays were used to detect apoptosis levels of circ-MAPK4 silenced U138 and U373 cells. (**g**) Western blot assays were performed to analyze the protein expression levels of cleaved form of caspase-9, caspase-7, caspase-3, and PARP1 in circ-MAPK4 silenced U138 and U373 cells. (**h**) Transwell assays were carried out to test the invasive activity of circ-MAPK4 silenced U138 and U373 cells. The results above were summarized as bar graph. All data are the means ± SEM of three experiments. *P < 0.05; **P < 0.01; ***P < 0.001

circ-MAPK4 was prominently (*P* < 0.05) expressed in glioma cell lines (especially U138 and U373) compared with glial cell line (HA1800) (Fig. 2a). To investigate the biological function of circ-MAPK4 in gliomas progression, we designed the circ-MAPK4 siRNAs against the back-spliced sequence of circ-MAPK4 (Fig. 2b). circ-MAPK4 siRNA was transfected into U138 and U373 cell lines which successfully silenced the circ-MAPK4 but not MAPK4 mRNA as confirmed by qPCR analysis (Fig. 2c). CCK-8 and colony formation assays were performed; inhibition of circ-MAPK4 caused a significant decrease in cells number (Fig. 2d, e). Meanwhile, cell cycle assay revealed that silencing of circ-MAPK4 had no effect on the cell cycle progression of glioma cells (Additional file 4: Figure S3). These results demonstrated that circ-MAPK4 markedly enhanced the survival of glioma cells but did not affect cell cycle progression.

To investigate further the biological function of circ-MAPK4, apoptosis of U138 and U373 cells was evaluated after transient transfection of the siRNAs followed by performing Annexin V-FITC/PI. The decline of circ-MAPK4 exhibited increased apoptosis in the experimental cells compared to control cells (Fig. 2f). Furthermore, the intracellular activities of cleaved form of caspase-9, caspase-7, caspase-3, and PARP1 significantly increased in circ-MAPK4 silencing cells compared with control cells (Fig. 2g). Transwell assays showed that the invasive ability was significantly inhibited after circ-MAPK4 was silenced in U138 and U373 cells (Fig. 2g).

**Fig. 3:**
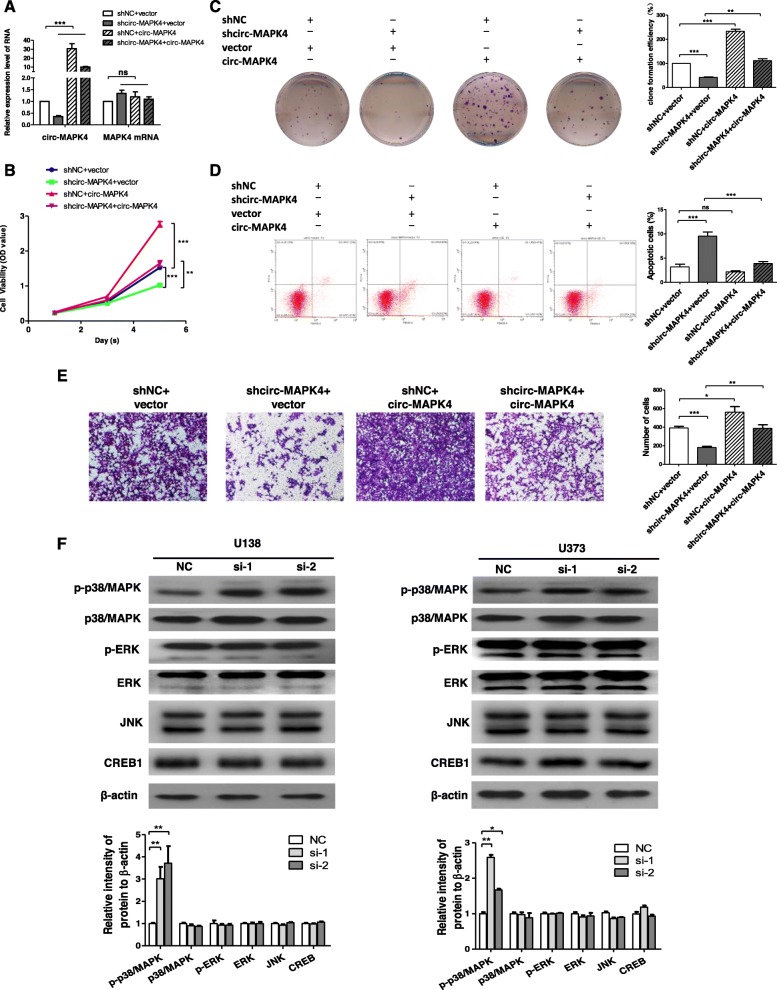
circ-MAPK4 maintains the survival of glioma cells via p38/MAPK pathway. (**a**) circ-MAPK4 expression was rescued in stable circ-MAPK4 silenced U138 cells. qPCR assay was performed to detect relative expression level of circ-MAPK4. (**b**, **c**) CCK-8 and colony formation assays were performed to test cell proliferation of these U138 cells. (**d**) Apoptosis assays were performed to test cellular apoptosis (ratio) of these U138 cells. (**e**) Transwell assays were performed to test cell invasive activity of these U138 cells with restored expression of circ-MAPK4. (**f**) Western blot assays were used to evaluate the total protein levels of p38/MAPK, ERK, JNK, CREB and the phosphorylation levels of p38/MAPK, ERK in transfected U138 and U373 cells. The intensity of bands were scanned and measured by Image J software, and were summarized as bar graphs. Data are the means ± SEM of three experiments. *P < 0.05; **P < 0.01; ***P < 0.001

To confirm further the oncogenic role of circ-MAPK4, expression of circ-MAPK4 was rescued in U138 cells which had circ-MAPK4 silenced. Firstly, qPCR assay showed increased expression level of circ-MAPK4 in sh-circ-MAPK4 U138 cells (Fig. 3a). Second, forced overexpression of circ-MAPK4 could significantly unmask the inhibition of cell survival (CCK-8 and cell clone formation assays) (Fig. 3b-c). Forced overexpression of circ-MAPK4 in these initially silenced U138 cells significantly reversed the level of apoptosis compared with the control group (Fig. 3d). Moreover, overexpression of circ-MAPK4 induced greater cell invasion (Fig. 3e). Taken together, the rescue experiments showed that circ-MAPK4 could maintain glioma cells survival, inhibit their apoptosis, and induce glioma cells invasion.

### Circ-MAPK4 maintains glioma cells survival via MAPK pathway

To identify the underlying mechanism that circ-MAPK4 inhibits apoptosis in glioma cells, we focused on the Mitogen Activated Protein Kinase (MAPK) signaling pathway including p38 kinases, c-Jun amino-terminal kinases (JNKs), and signal-regulated kinases (ERKs), which are crucial molecules involved in cancer pathogenesis. We performed western blot assays to determine protein expression levels of MAPK signaling pathway in circ-MAPK4 silenced glioma cells. Higher phosphorylation level of p38/MAPK (p-p38/MAPK) was observed in circ-MAPK4 silenced glioma cells compared with control cells, while total protein levels of p38, ERK, JNK, CREB and the phosphorylation level of ERK remained unchanged (Fig. 3f). Base on the proposed tumor suppressor role of p38/MAPK, phosphorylation of p38/MAPK will activate its effect in inhibiting cell proliferation, which is consistent with what’s shown and what happens with circ-MAPK4 silencing. Therefore, defects in p38/MAPK function may contribute to oncogenic effect of circ-MAPK4 in glioma cells.

**Fig. 4:**
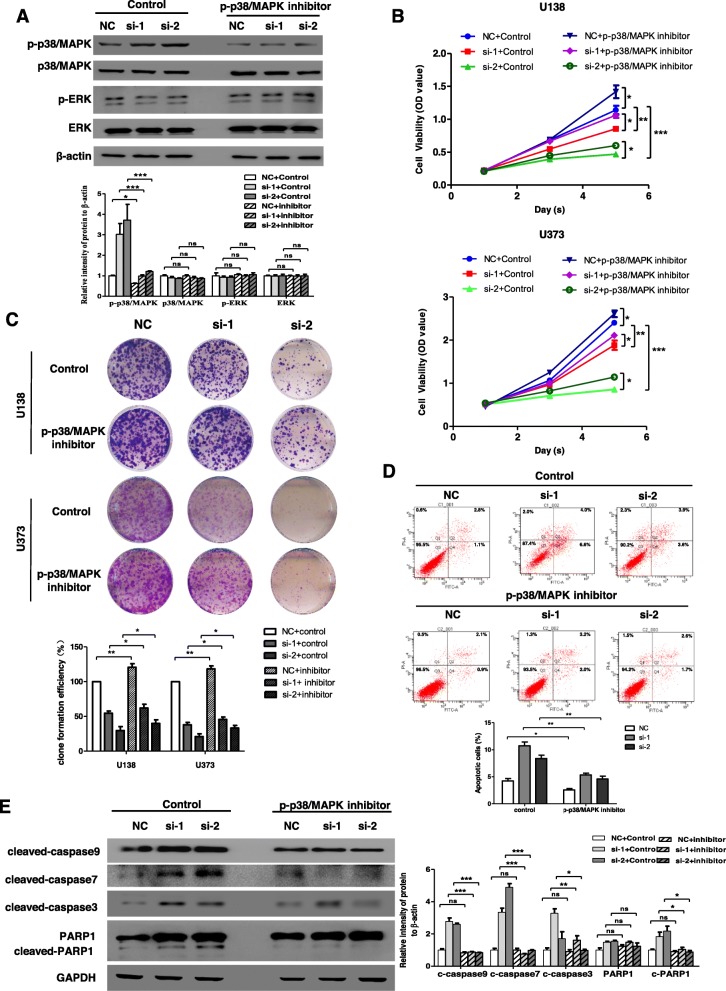
Inhibition of phosphorylation of p38/MAPK reverses cell survival induced by circ-MAPK4. (**a**) U373 cells transfected with siRNA negative control, circ-MAPK4 siRNA-1 and siRNA-2 were treated with or without p-p38/MAPK inhibitor (SB203580). Inhibition efficiency of p-p38/MAPK inhibitor was accessed by testing phosphorylation and total protein levels of p38/MAPK and ERK using western blot assays. (**b**, **c**) CCK-8 and colony formation assays were performed to reveal cell survival of these U373 cells. (**d**) Apoptosis assays were performed to reveal apoptosis levels of these U373 cells. (**e**) The western blot assays were used to evaluate effect of p-p38/MAPK inhibitor on protein expression levels of cleaved form of caspase-9, caspase-7, caspase-3, PARP1 in these U373 cells. The data were summarized as bar graph. Data are the means ± SEM of three experiments. *P < 0.05; **P < 0.01; ***P < 0.001

To prove further that p38/MAPK pathway was necessary for circ-MAPK4 function, we used p-p38/MAPK inhibitor (SB203580) to block phosphorylation levels of p38/MAPK in U138 and U373 cells which were initially transfected with either si-circ-MAPK4 or siNC. Compared with the control group, SB203580 indeed inhibited the phosphorylation levels of p38/MAPK, while the phosphorylation levels of ERK did not change (Fig. 4a). CCK-8 and cell colony formation assays confirmed that SB203580 which blocked phosphorylation of p38/MAPK unmasked the proliferative potential abrogated by silencing circ-MAPK4, and leaded to more cell viability compared with the control group (Fig. 4b, c). Furthermore, SB203580 leaded to a 5.4 and 3.8% reduction of apoptosis ratio in circ-MAPK4 silenced U373 cells compared with the control group (Fig. 4d). Western blot assay further showed that the increased levels of cleaved form of apoptosis effectors caspase-3, caspase-9, caspase-7 and PARP1 were significant decreased in the p-p38/MAPK inhibitor group versus control group (Fig. 4e). But the p-p38/MAPK inhibitor had no effect on reversing the function of circ-MAPK4 in the invasive ability of glioma cells (Additional file 5: Figure S4), which suggested that circ-MAPK4 might regulate cell invasion through another signaling pathway. Taken together, circ-MAPK4 was proved to maintain cell survival through blocking p38/MAPK signaling pathway in vitro.

### miR-125a-3p is the effector of circ-MAPK4 in regulating p38/MAPK signaling pathway

**Fig. 5:**
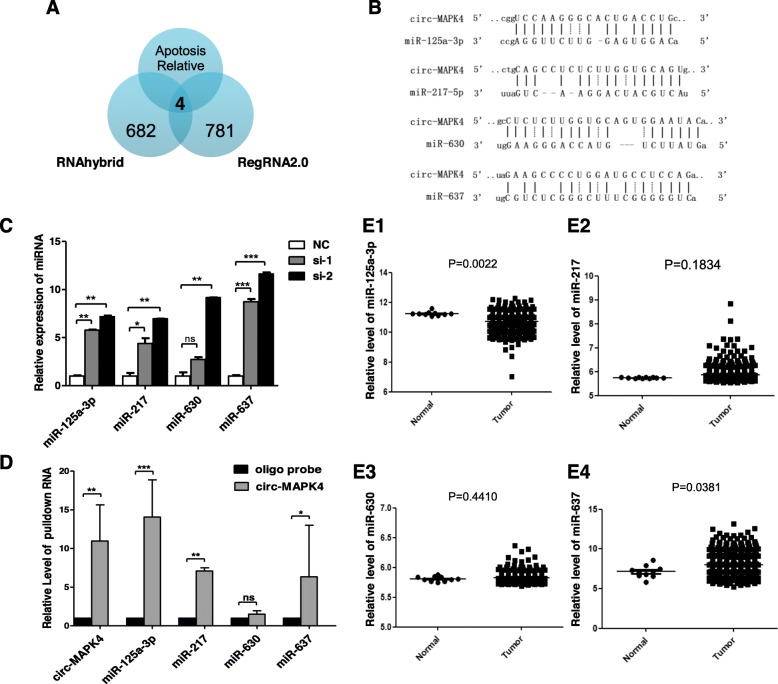
circ-MAPK4 acts as a sponge of miR-125a-3p in glioma cells. (**a**) Schematic illustration showing the overlap of target miRNAs of circ-MAPK4 predicted by RegRNA1.0, RNAhybrid and proved apoptosis relative miRNAs. (**b**) Predicted complementary binding sites between circ-MAPK4 and miRNAs. (**c**) qPCR were performed to evaluate expression levels of four predicted target miRNAs in circ-MAPK4 silenced U138 cells. (**d**) miRNAs were pulled down by the biotin labeled probe targeting to either random sequences or circ-MAPK4 black-splice junction sequence. Enrichment of the four predicted miRNAs was analyzed using qPCR. (E1–4) Relatively differential expression levels of the four predicted miRNAs between glioma tissues and normal brain tissues were analyzed based on the TCGA data

**Fig. 6:**
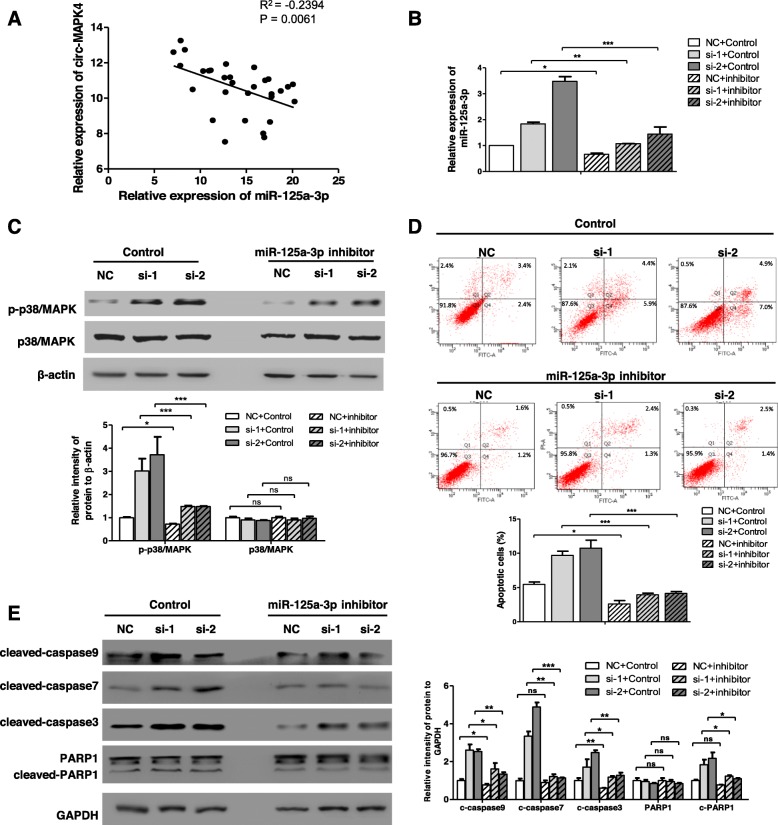
circ-MAPK4 regulates p38/MAPK4 signaling pathway by targeting miR-125a-3p. (**a**) Expression of circ-MAPK4 and miR-125a-3p are inversely correlated in glioma tissues (R^2^ = -0.2394, *P* = 0.0061). (**b**) qPCR was performed to test silence effect of the miR-125a-3p inhibitor. (**c**) Western blot assays evaluated phosphorylation and total protein levels of p38/MAPK in circ-MAPK4 silenced U373 cells which were treated either with or without miR-125a-3p inhibitor. (**d**, **e**) Apoptosis and western blot assays revealed levels of cellular apoptosis and apoptosis effectors after inhibiting miR-125a-3p in circ-MAPK4 silenced U373 cells. Intensity of bands were scanned and measured by Image J software, and summarized as bar graph. All experiments were performed at least three times. Data are presented as the mean ± SD. *P < 0.05; **P < 0.01; ***P < 0.001

circRNAs may act as miRNA ‘sponges’ because of the presence of complementary binding sites with miRNAs. Hence, we utilized the bioinformatics program (RegRNA2.0, http://regrna.mbc.nctu.edu.tw, and RNAhybrid, https://bibiserv.cebitec.uni-bielefeld.de/rnahybrid) to predict and filter possible miRNA candidates. Combined with the expected function in inducing glioma cells apoptosis, four miRNAs (Fig. 5a) (miR-125a-3p, miR-217, miR-630, and miR-637) were chosen, which shared binding sites with circ-MAPK4 (Fig. 5b). Expression levels of these four candidate miRNAs increased in U138 cells when these cells were transfected with si-circ-MAPK4 (Fig. 5c). Pull-down assay combined with qPCR analysis indicated that circ-MAPK4 could specifically bind these miRNAs, especially miR-125a-3p (*P* < 0.001) (Fig. 5d). Expression levels of these four candidate miRNAs in 565 gliomas and 10 normal brain tissues were tested from miRNA-microarray data (The Cancer Genome Atlas (TCGA, https:// cancergenome.nih.gov/). miR-125a-3p was significantly lower in gliomas compared with normal brain tissues; whereas no significant differences between gliomas and normal brain tissues in level of miR-217 and miR-630. miR-637 expression was lower in normal brain tissues compared to gliomas (Fig. 5e1-e4). Taken together, miR-125a-3p is a candidate target molecule of circ-MAPK4. Furthermore, a significant inverse correlation occurred between circ-MAPK4 and miR-125a-3p in glioma tissues (R squared = 0.2394, *P* < 0.01) (Fig. 6a). But overexpression of circ-MAPK4 in U373 cells did not directly induce the downregulation of miR-125a-3p (Additional file 6: Figure S5). Therefore, we suggested that miR-125a-3p was regulated but not degraded by circ-MAPK4 in gliomas.

To understand the interaction of circ-MAPK4 and miR-125a-3p in regulating p38/MAPK signaling pathway, we used a miR-125a-3p inhibitor to block the induced expression in the initially circ-MAPK4 silenced U373 cells. qPCR result showed that miR-125a-3p inhibitor indeed decreased expression of miR-125a-3p in U373 (Fig. 6b). Phosphorylation of p38/MAPK was partly rescued by miR-125a-3p inhibitor compared with the control group (Fig. 6c). Further, the increase in apoptosis (ratio) and apoptosis-related proteins caused by circ-MAPK4 silencing were rescued by the miR-125a-3p inhibitor compared with the control group (Fig. 6d, e). In summary, we determined that circ-MAPK4 regulated cell apoptosis of glioma cells through p38/MAPK signaling pathway by sponging miR-125a-3p.

### Circ-MAPK4 promotes the tumorigenesis in vivo

**Fig. 7:**
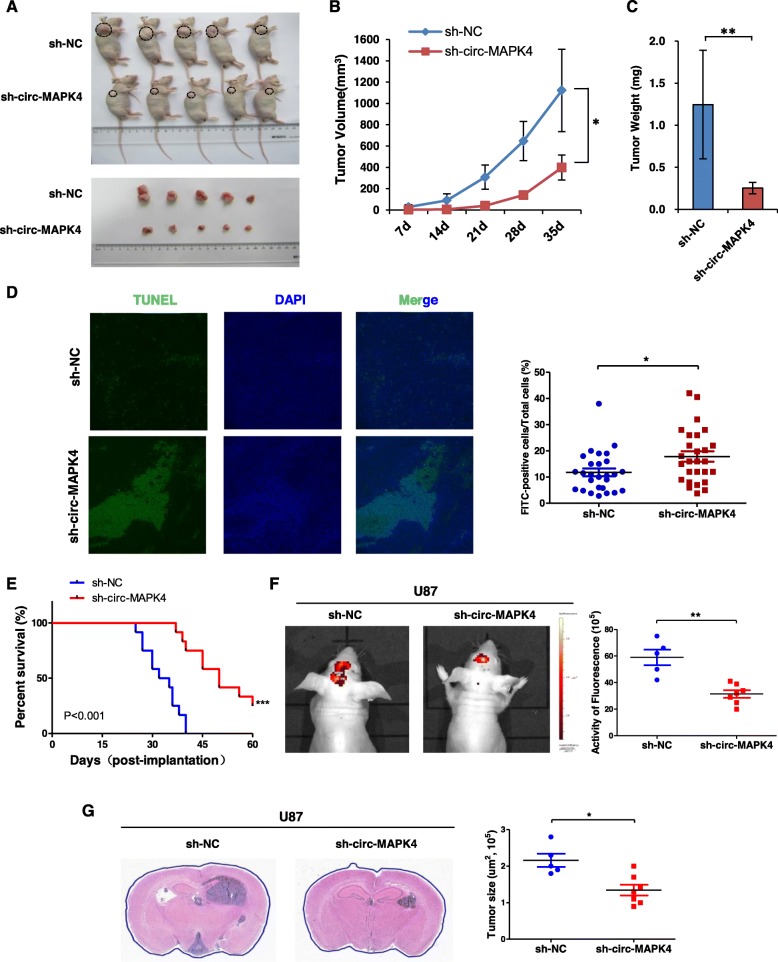
circ-MAPK4 promotes the tumorigenesis in vivo. (**a**) A total of 10 male BALB/c nude mice (*n* = 5 each group) were injected with either the stable circ-MAPK4 silenced or parent control U138 cells (5 × 10^6^). Nude mice were sacrificed after five weeks and the xenografted tumors were measured and cut. (**b**) Sizes of xenografted tumors were measured every 7 days. (**c**) Weights of xenografted tumors were summarized as a bar graph. (**d**) TUNEL assays were performed to detect the cellular apoptosis of xenografted tumors. Three xenografted tumors of each group were randomly choosed and three discontinuous sections were selected to perform the TUNEL assays. After randomly selecting three fields of view from each section, the FITC-positive cells were counted and stable circ-MAPK4 silenced group was compared to control group. (**e**) Survival curve of shRNA negative control (blue line) and circ-MAPK4 silenced U87 injected mice (red line; Kaplan-Meier plots). Survival was significantly increased in circ-MAPK4 silenced group (P < 0.001). (**f**) In vivo tumorigenicity was monitored. Downregulation of circ-MAPK4 significantly inhibited the formation of brain tumors. (**g**) GBM areas (μm^2^) of each tumor were measured, and experimental group was significantly lower than control group. *P < 0.05, **P < 0.01, ***P < 0.001

In vivo, U138 cells were stably transfected with shNC or sh-circ-MAPK4 plasmid with GFP marker. Either stable circ-MAPK4 knockdown or negative control U138 cells (5 × 10^6^ cells) were subcutaneously injected in BALB/c nude mice. Tumors were allowed to grow for 35 days, and tumor volumes were measured every 7 days. Silencing of circ-MAPK4 generated notable decrease in rate and tumor size of xenografts (Fig. 7a-c). The relative expression of circ-MAPK4 and miR-125a-3p using qPCR in ten tumors collected from ectopic xenograft study is shown on Additional file 7: Figure S6A. We found that the expression level of circ-MAPK4 was positively correlated with the size of ectopic tumors, while the expression level of miR-125a-3p was negatively correlated with the size of the ectopic tumors (Additional file 7: Figure S6B). TUNEL assay also showed that knockdown of circ-MAPK4 induced apoptosis of glioma cells compared to the control group (*P* < 0.05) (Fig. 7d).

To confirm further the role of circ-MAPK4 in brain tumorigenesis, orthotopic xenografts were performed using BALB/c nude mice. Nude mice (*n* = 12 each group) were intracranially injected with either stable circ-MAPK4 silenced or negative control U87 cells. Survival analysis was calculated by Kaplan-Merier curve, circ-MAPK4 silencing significantly increased the overal survival time of orthotopic mouse model (*P* < 0.001). The circ-MAPK4 silenced mice lived 50 days, whereas the control group lived 34 days (median survival; Fig. 7e). In vivo tumorigenicity monitored by In-Vivo FX Pro system every 5 days showed that downregulation of circ-MAPK4 significantly inhibited formation of xenograft brain tumors (activity of fluorescence, *p* < 0.05) (Fig. 7f). GBM area (10^5^, μm^2^) of each tumor was calculated, and experimental group was significantly lower than control group (P < 0.05) (Fig. 7g). Taken together, circ-MAPK4 can enhance survival of glioma cells through inhibiting cell apoptosis both in vitro *and* in vivo.

## Discussion

Growing evidence indicates that noncoding RNAs participate in glioma carcinogenesis [20]. CircRNAs, are a subclass of noncoding RNAs with high conservation and very stable circular structure, making them ideal biomarkers for diagnosis of disease. For instance, circTTBK2 is highly expressed in glioma, promotes glioma cell metastasis in vitro, and is a potential prognostic tumor marker for gliomas [21]. Another, circMMP9 induced by eIF4A3 enhances cell proliferation, invasion and metastasis of GBM through modulation of the miR-124 signaling pathway, which could provide pivotal potential therapeutic targets for treatment of GBM [16]. Here, we showed that a circRNA, circ-MAPK4 is positively correlated with survival of glioma cells by inhibiting their apoptosis.

We show that circ-MAPK4 is significantly overexpressed in gliomas compared with the non-tumor brain tissues. At the same time, circ-MAPK4 is downregulated during early stage of neural differentiation. To the least of our knowledge, this is the first time to scan circRNAs which play an oncogene role in cancer using development model. Cancer cells are dedifferentiated compared to their normal counterpart. Exploring this concept within the context of circRNA, we scanned for downregulated circRNAs upon inducing neural differentiation of murine P19 embryonic carcinoma (EC). Circ-MAPK4 is downregulated during neural differentiation; hence, we hypothesized that it can play a role in the development and progression of gliomas. In experiments, silencing of circ-MAPK4 significantly inhibited survival of glioma cells. In addition, flow cytometry analysis indicated that circ-MAPK4 downregulation inhibited glioma cells survival by inducing cell apoptosis.

Apoptosis is a programmed cellular suicide mechanism that maintains cellular homeostasis. However, by amplifying anti-apoptotic machinery and/or downregulating pro-apoptotic programs, cancer cells can override apoptosis and promote progression of disease. In this study, silencing of circ-MAPK4 caused levels of the cleaved form of apoptosis effectors (caspse-9, caspase-7, caspase-3 and PARP) increased, suggesting the anti-apoptotic role of circMAPK4 in gliomas progression.

Considering of the host mRNA of circ-MAPK4, MPAK4, is a member of the mitogen-activated protein kinase family. We tested whether circ-MAPK4 can impact the MAPK pathway. Four signaling pathways of the MAPK family have been identified in eukaryotic cells: (i) Extracellular signal-regulated protein kinase (ERK); (ii) c-Jun N-terminal kinase (JNK)/stress-activated protein kinase (SAPK); (iii) ERK5/big MAP kinase (BMK1); and (iv) p38/MAPK pathways [22]. The p38/MAPK pathway has been widely demonstrated to act in the pathophysiological processes of cell differentiation, development and regulation of apoptosis [23]. P38/MAPK activation was previously identified to induce apoptosis in non-tumor cells, such as nerve cells [24], fetal brown adipocytes [25] and tumor cells [26–28]. Studies have identified p38/MAPK to be an independent effector of inducing cellular apoptosis [29, 30]. However, controversy exists whether p38/MAPK is a positive- or negative-regulator of apoptosis [31]. In this study, we found that inhibition of circ-MAPK4 increases the phosphorylation levels of p38/MAPK, at the same time, increased levels of cellular apoptosis. In contrast, phosphorylation levels of ERK、JNK did not change. Furthermore, we found that a p38/MAPK inhibitor can reverse cellular apoptosis potential inducing by silencing circ-MAPK4. All the results suggest the role of circ-MAPK4 is to downregulate phosphorylation levels of p38/MAPK, which inhibits apoptosis of glioma cells.

According to DIANA microT-CDS database and other studies, miR-125a-3p is downregulated in malignant gliomas. Its role as tumor suppressor was confirmed by overexpressing miR-125a-3p which induced markedly apoptosis and suppressed proliferation and migration of glioblastoma cells in vitro *and* in vivo [19]. Our results showed that miR-125a-3p was pulled down by circ-MAPK4 when doing immunoprecipitation studies. This is consistent with the idea that circRNA acts not as a “sponge” but as a “boat” to prevent its passengers from drowning and can move on to new ports [32]. The upregulated circ-MAPK4 “loads” miR-125a-3p to prevent its binding with target oncogenes in gliomas. Our result also showed that miR-125a-3p was upregulated in U373 cells where circ-MAPK4 was silenced. However, in the gain of function experiments, overexpression of circ-MAPK4 did not induce a decline of miR-125a-3p, suggesting that circ-MAPK4 could govern miR-125a-3p but did not result in the degradation of miR-125a-3p. Similar to siRNA silencing of its target mRNA, a perfect reverse complementary is needed between circRNA and miRNA to induce degradation. We hypothesized that the upregulation of miR-125a-3p after silencing circ-MAPK4 occurred by other signaling pathways impacted by circ-MAPK4. The promoter of miR-125a is activated by NF-κB [33], which can be activated by the MAPK signaling pathway [34]. Hence, we hypothesize that activation of MAPK signaling pathway is induced by the silencing of circ-MAPK4, which initiates the downstream induction of NF-κB which will increase the activity of the promoter of miR-125a. This hypothesis requires more studies.

Considering the role of circ-MAPK4 in regulating phosphorylation levels of p38/MAPK, we were interested in whether miR-125-3p has the same function. Consistent with our hypothesis, we found that the phosphorylation level of p38/MAPK can be reversed by a miR-125a-3p inhibitor in U373 cells after circ-MAPK4 has been silenced, levels of phosphorylation of ERK、JNK did not change. Perhaps circ-MAPK4 targets miR-125a-3p, which helps control p38/MAPK signaling pathway to regulate cellular apoptosis. However, miR-125a-3p may not directly regulate the phosphorylation levels of p38/MAPK. Further studies are needed.

In conclusion: 1.We identify a novel circ-RNA (circ-MAPK4), which is downregulated during neural differentiation and upregulated in glioma tissues. 2. We verify that circ-MAPK4 is generated from exon 2 of MAPK4 mRNA, and acts as oncogene to promote glioma cell survival and to inhibit apoptosis. 3. We suggest that apoptosis of glioma cancer cells is inhibited by circ-MAPK4 through downregulation of phosphorylation of p38/MAPK. 4. We demonstrate that miR-125a-3p is the effector of circ-MAPK4 in regulating of p38/MAPK pathway. 5. We verify that circ-MAPK4 can promote tumorigenesis in vivo. Therefore, we suggest that circ-MAPK4 may be a promising biomarker and therapeutic target for gliomas.

## Supplementary information

**Additional file 1: Table S1.** The primer sequences for the qRT-PCR. **Table S2.** siRNA sequence targeting circ-MAPK4. **Table S3.** The DNA oligo sequences for pull-down assays

**Additional file 2: Figure S1.** A. MiOncoCirc database showed that circ-MAPK4 was upregulation in GBM. B. qPCR assays were performed to examine the expression profile of other 9 circRNAs which were downregulated in neural differentiation model.

**Additional file 3: Figure S2.** A. Sequences of circ-MAPK4 were highly conservative among mammals and 46 vertebrate species (upper panel). The Conserved Elements were also measured to identify the main conservative sites among mammals and 46 vertebrate species (bottom panel). B. Multiple alignments of 10 flanking sequences of 5′- and 3′-end showed that the core sequences mediating head-to-tail splicing of junction site of circ-MAPK4 were highly conserved among 46 vertebrate species

**Additional file 4: Figure S3.** Cell cycle progression of the glioma cells after silencing of circ-MAPK4. Glioma cells (U138) were transfected with circ-MAPK4 siRNAs, and cell cycle assays was performed to test the impact of circ-MAPK4 on progression of the cell cycle. Experiments were repeated three times. All results are summarized on a graph bar and presented as means ± standard deviation (SD)

**Additional file 5: Figure S4.** Tanswell assay proposed that p-p38/MAPK inhibitor had no effect on reversing the function of circ-MAPK4 on enhancing invasive ability of glioma cancer cells

**Additional file 6: Figure S5.** qPCR assays showed that overexpression of circ-MAPK4 in U373 cells did not induce degradation of miR-125a-3p

**Additional file 7: Figure S6.** A. qPCR assays measure the relative expression levels of circ-MAPK4 and miR-125a-3p in ten tumors collected from ectopic xenograft study. B. Expression levels of circ-MAPK4 and miR-125a-3p correlate with the sizes of ectopic tumors

## Data Availability

Not applicable.

## Abbreviations

CircRNA: Circular RNA
CNS: Central nervous system
GBM: Glioblastoma multiforme
MAPK: Mitogen Activated Protein Kinase
MiRNA: MicroRNA
NcRNA: Noncoding RNA
P-p38: Phosphorylation of p38
SiRNA: Small interfering RNA
