# Supplementary figures and images for: Circular RNA MAPK4 (circ-MAPK4) inhibits cell apoptosis via MAPK signaling pathway by sponging miR-125a-3p in gliomas
Source: Mol Cancer. 2020 Jan 28;19:17. doi: 10.1186/s12943-019-1120-1 (PMC6986105; doi:10.1186/s12943-019-1120-1)

**A**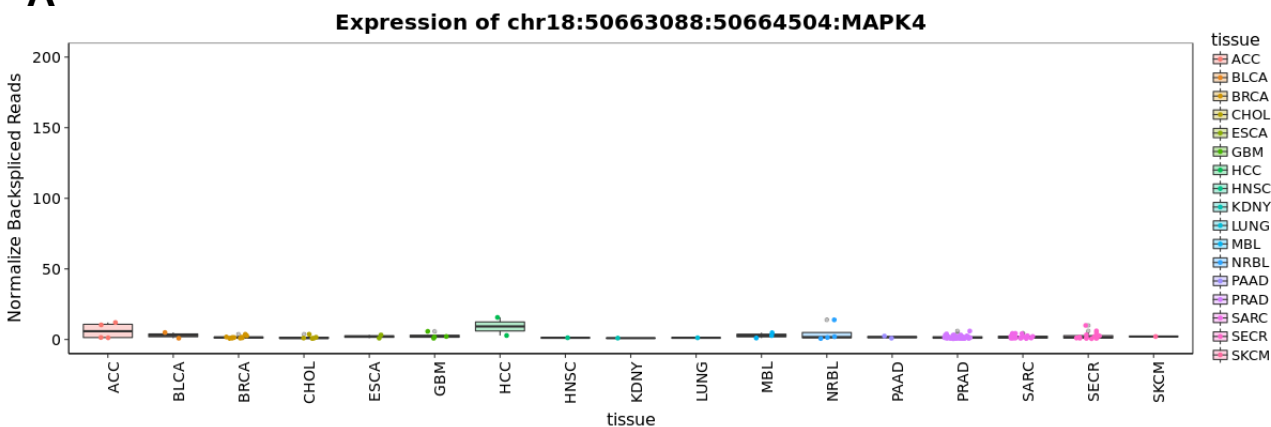**B**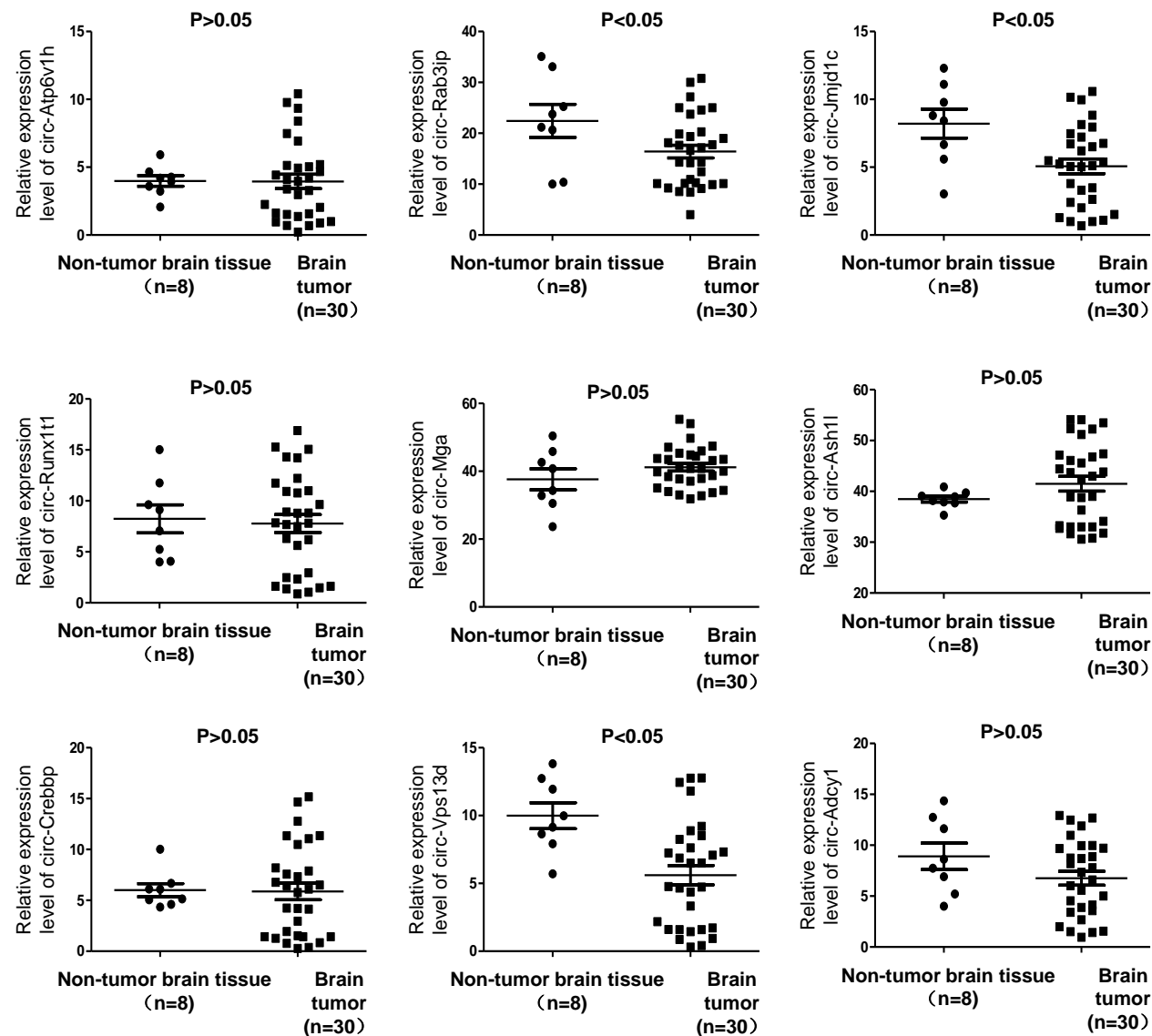

Supplement: Supplementary file 2 — Additional file 2: Figure S1. A. MiOncoCirc database showed that circ-MAPK4 was upregulation in GBM. B. qPCR assays were performed to examine the expression profile of other 9 circRNAs which were downregulated in neural differentiation model. [file 12943_2019_1120_MOESM2_ESM.pdf]

# U138

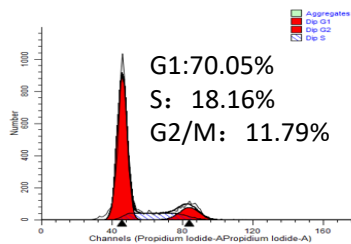

NC

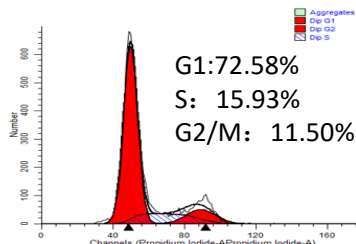

si-1

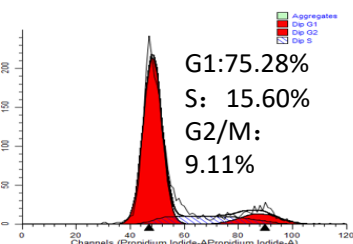

si-2

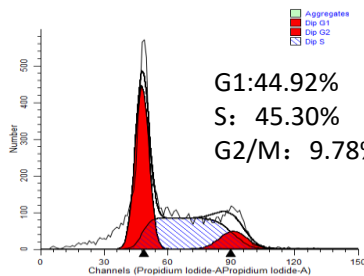

NC

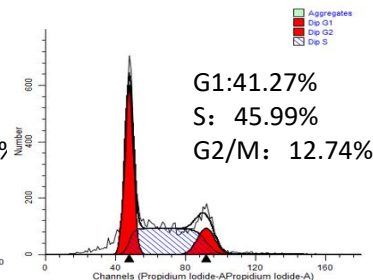

si-1

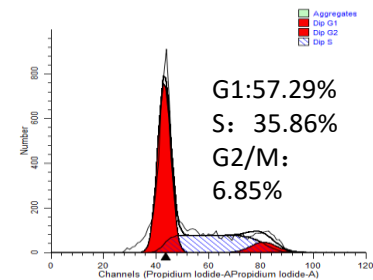

si-2

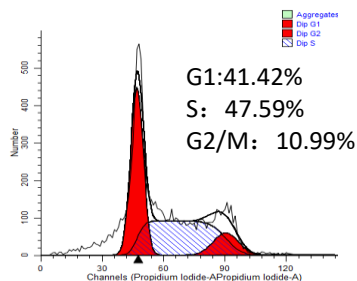

NC

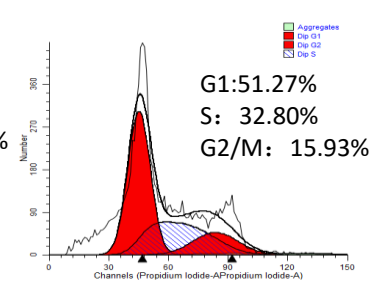

si-1

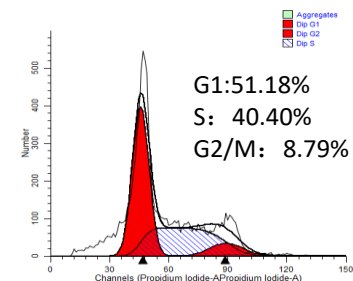

si-2

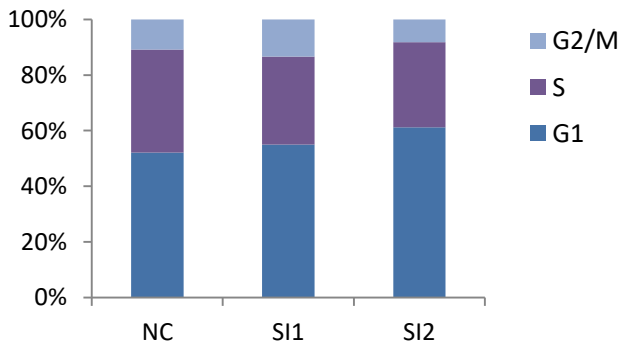

Supplement: Supplementary file 4 — Additional file 4: Figure S3. Cell cycle progression of the glioma cells after silencing of circ-MAPK4. Glioma cells (U138) were transfected with circ-MAPK4 siRNAs, and cell cycle assays was performed to test the impact of circ-MAPK4 on progression of the cell cycle. Experiments were repeated three times. All results are summarized on a graph bar and presented as means ± standard deviation (SD) [file 12943_2019_1120_MOESM4_ESM.pdf]

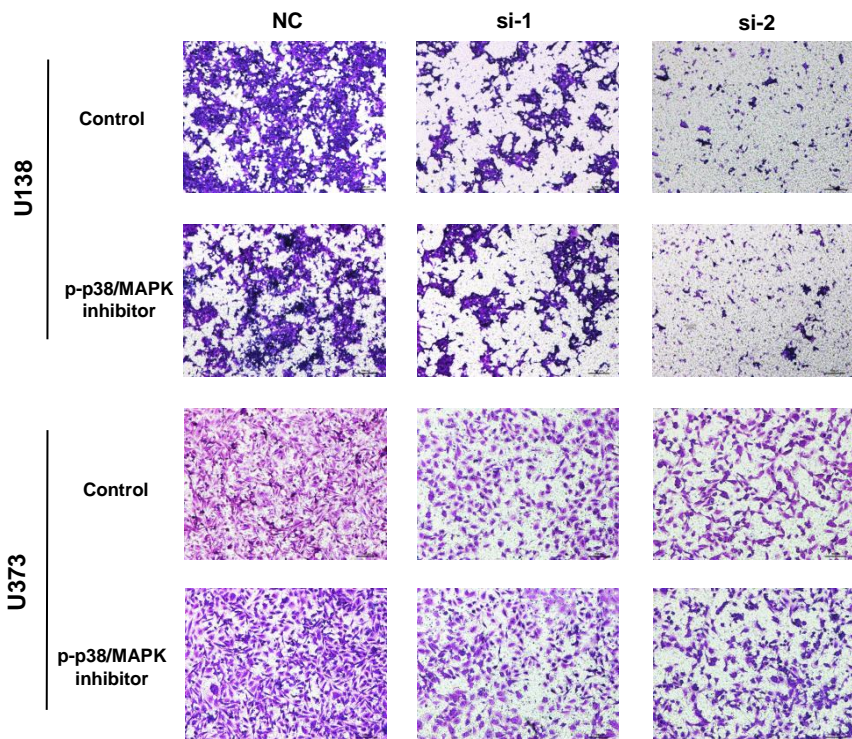

Supplement: Supplementary file 5 — Additional file 5: Figure S4. Tanswell assay proposed that p-p38/MAPK inhibitor had no effect on reversing the function of circ-MAPK4 on enhancing invasive ability of glioma cancer cells [file 12943_2019_1120_MOESM5_ESM.pdf]

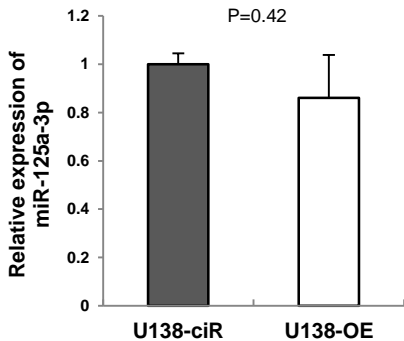

Supplement: Supplementary file 6 — Additional file 6: Figure S5. qPCR assays showed that overexpression of circ-MAPK4 in U373 cells did not induce degradation of miR-125a-3p [file 12943_2019_1120_MOESM6_ESM.pdf]

**A**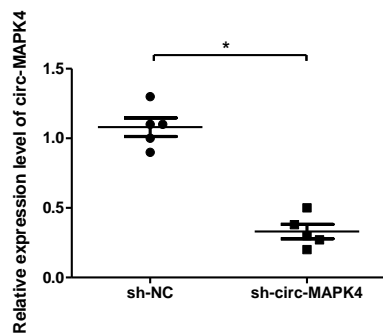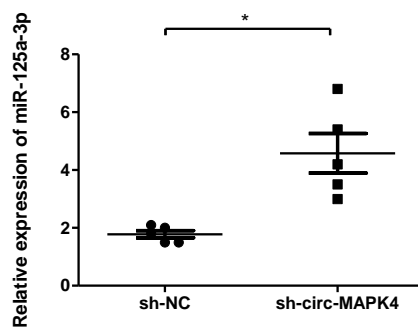**B**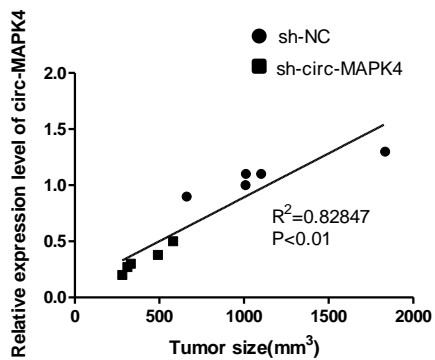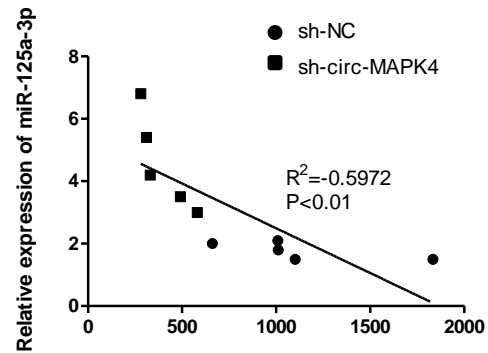

Supplement: Supplementary file 7 — Additional file 7: Figure S6. A. qPCR assays measure the relative expression levels of circ-MAPK4 and miR-125a-3p in ten tumors collected from ectopic xenograft study. B. Expression levels of circ-MAPK4 and miR-125a-3p correlate with the sizes of ectopic tumors [file 12943_2019_1120_MOESM7_ESM.pdf]
