# Supplementary material for: Circular RNA MAPK4 (circ-MAPK4) inhibits cell apoptosis via MAPK signaling pathway by sponging miR-125a-3p in gliomas
Source: Mol Cancer. 2020 Jan 28;19:17. doi: 10.1186/s12943-019-1120-1 (PMC6986105; doi:10.1186/s12943-019-1120-1)
Supplement: Supplementary file 3 — Additional file 3: Figure S2. A. Sequences of circ-MAPK4 were highly conservative among mammals and 46 vertebrate species (upper panel). The Conserved Elements were also measured to identify the main conservative sites among mammals and 46 vertebrate species (bottom panel). B. Multiple alignments of 10 flanking sequences of 5′- and 3′-end showed that the core sequences mediating head-to-tail splicing of junction site of circ-MAPK4 were highly conserved among 46 vertebrate species [file 12943_2019_1120_MOESM3_ESM.pdf]

A

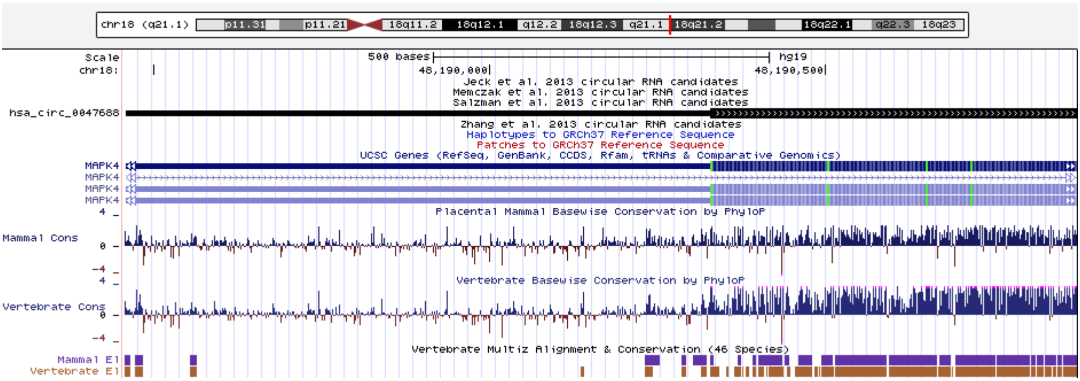
chr18 (q21.1)
p11.31
p11.21
18q11.2
18q12.1
q12.2
18q12.3
q21.1
18q21.2
18q22.1
q22.3
18q23
Scale
chr18:
500 bases
hg19
48,190,000
48,190,500
Jeck et al. 2013 circular RNA candidates
Memczak et al. 2013 circular RNA candidates
Salzman et al. 2013 circular RNA candidates
hsa_circ_0047688
Zhang et al. 2013 circular RNA candidates
Haplotypes to GRCh37 Reference Sequence
Patches to GRCh37 Reference Sequence
UCSC Genes (RefSeq, GenBank, CCDS, Rfam, tRNAs & Comparative Genomics)
MAPK4
MAPK4
MAPK4
MAPK4
Placental Mammal Basewise Conservation by PhyloP
Mammal Cons
4
0
-4
Vertebrate Basewise Conservation by PhyloP
Vertebrate Cons
4
0
-4
Vertebrate Multiz Alignment & Conservation (46 Species)
Mammal El
Vertebrate El

B

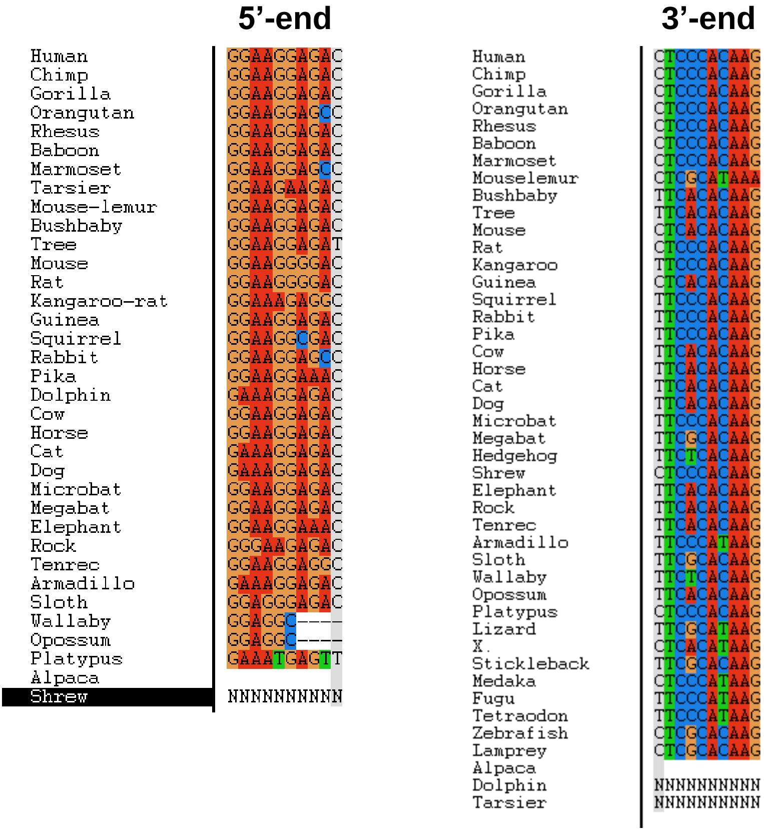
5'-end
Human GGAAGGAGAC
Chimp GGAAGGAGAC
Gorilla GGAAGGAGAC
Orangutan GGAAGGAGCC
Rhesus GGAAGGAGAC
Baboon GGAAGGAGAC
Marmoset GGAAGGAGCC
Tarsier GGAAGAAGAC
Mouse-lemur GGAAGGAGAC
Bushbaby GGAAGGAGAC
Tree GGAAGGAGAT
Mouse GGAAGGGGAC
Rat GGAAGGGGAC
Kangaroo-rat GGAAAGAGGC
Guinea GGAAGGAGAC
Squirrel GGAAGGCGAC
Rabbit GGAAGGAGCC
Pika GGAAGGAAAC
Dolphin GAAAGGAGAC
Cow GGAAGGAGAC
Horse GGAAGGAGAC
Cat GAAAGGAGAC
Dog GAAAGGAGAC
Microbat GGAAGGAGAC
Megabat GGAAGGAGAC
Elephant GGAAGGAAAC
Rock GGGAAGAGAC
Tenrec GGAAGGAGGC
Armadillo GAAAGGAGAC
Sloth GGAGGGAGAC
Wallaby GGAGGC----
Opossum GGAGGC----
Platypus GAAATGAGTT
Alpaca
Shrew NNNNNNNNNN
3'-end
Human CTCCCACAAG
Chimp CTCCCACAAG
Gorilla CTCCCACAAG
Orangutan CTCCCACAAG
Rhesus CTCCCACAAG
Baboon CTCCCACAAG
Marmoset CTCCCACAAG
Mouselemur CTCGCATAAA
Bushbaby TTCACACAAG
Tree TTCACACAAG
Mouse CTCACACAAG
Rat CTCCCACAAG
Kangaroo TTCCCACAAG
Guinea CTCACACAAG
Squirrel TTCCCACAAG
Rabbit TTCCCACAAG
Pika TTCCCACAAG
Cow TTCACACAAG
Horse TTCACACAAG
Cat TTCACACAAG
Dog TTCACACAAG
Microbat TTCCCACAAG
Megabat TTCGCACAAG
Hedgehog TTCTCACAAG
Shrew CTCCCACAAG
Elephant TTCACACAAG
Rock TTCACACAAG
Tenrec TTCACACAAG
Armadillo TTCCCATAAG
Sloth TTCGCACAAG
Wallaby TTCTCACAAG
Opossum TTCACACAAG
Platypus CTCCCACAAG
Lizard TTCGCATAAG
X. CTCACATAAG
Stickleback TTCGCACAAG
Medaka CTCCCATAAG
Fugu TTCCCATAAG
Tetraodon TTCCCATAAG
Zebrafish CTCGCACAAG
Lamprey CTCGCACAAG
Alpaca
Dolphin NNNNNNNNNN
Tarsier NNNNNNNNNN
